# FoxM1 Promotes Stemness and Radio-Resistance of Glioblastoma by Regulating the Master Stem Cell Regulator Sox2

**DOI:** 10.1371/journal.pone.0137703

**Published:** 2015-10-07

**Authors:** Yeri Lee, Kang Ho Kim, Dong Geon Kim, Hee Jin Cho, Yeonghwan Kim, Jinguen Rheey, Kayoung Shin, Yun Jee Seo, Yeon-Sook Choi, Jung-Il Lee, Jeongwu Lee, Kyeung Min Joo, Do-Hyun Nam

**Affiliations:** 1 Department of Health Sciences and Technology, SAIHST, Sungkyunkwan University, Seoul, South Korea; 2 Samsung Biomedical Research Institute, Samsung Medical Center, Seoul, South Korea; 3 Department of Neurosurgery, Samsung Medical Center, Sungkyunkwan University School of Medicine, Seoul, South Korea; 4 Department of Anatomy and cell biology, Sungkyunkwan University School of Medicine, Suwon, South Korea; 5 Department of Stem Cell Biology and Regenerative Medicine, Lerner Research Institute, Cleveland Clinic, Cleveland, OH, United States of America; Swedish Neuroscience Institute, UNITED STATES

## Abstract

Glioblastoma (GBM) is the most aggressive and most lethal brain tumor. As current standard therapy consisting of surgery and chemo-irradiation provides limited benefit for GBM patients, novel therapeutic options are urgently required. Forkhead box M1 (FoxM1) transcription factor is an oncogenic regulator that promotes the proliferation, survival, and treatment resistance of various human cancers. The roles of FoxM1 in GBM remain incompletely understood, due in part to pleotropic nature of the FoxM1 pathway. Here, we show the roles of FoxM1 in GBM stem cell maintenance and radioresistance. ShRNA-mediated FoxM1 inhibition significantly impeded clonogenic growth and survival of patient-derived primary GBM cells with marked downregulation of Sox2, a master regulator of stem cell phenotype. Ectopic expression of Sox2 partially rescued FoxM1 inhibition-mediated effects. Conversely, FoxM1 overexpression upregulated Sox2 expression and promoted clonogenic growth of GBM cells. These data, with a direct binding of FoxM1 in the Sox2 promoter region in GBM cells, suggest that FoxM1 regulates stemness of primary GBM cells via Sox2. We also found significant increases in FoxM1 and Sox2 expression in GBM cells after irradiation both *in vitro* and *in vivo* orthotopic tumor models. Notably, genetic or a small-molecule FoxM1 inhibitor-mediated FoxM1 targeting significantly sensitized GBM cells to irradiation, accompanying with Sox2 downregulation. Finally, FoxM1 inhibition combined with irradiation in a patient GBM-derived orthotopic model significantly impeded tumor growth and prolonged the survival of tumor bearing mice. Taken together, these results indicate that the FoxM1-Sox2 signaling axis promotes clonogenic growth and radiation resistance of GBM, and suggest that FoxM1 targeting combined with irradiation is a potentially effective therapeutic approach for GBM.

## Introduction

Glioblastoma (GBM) is the most common and lethal primary brain tumor. Currently, the standard-of-care treatment for GBM patients consists of surgical resection followed by radiation and chemotherapy. Despite these maximal therapies, the median survival of GBM patients is still only 14.6 months.[1] Therapeutic benefit of irradiation and TMZ treatments is only transient, due in most part to the resistance mechanisms elicited by GBM. Novel therapeutic approaches that can target core oncogenic pathways and/or pathways that confer treatment resistance to tumor cells are urgently needed.

As GBM’s former full name “Glioblastoma Multiforme” refers to, GBM tumor cells reveal highly heterogeneous morphologies and biological properties. A series of recent reports showed that multiple clones with distinct genomic alterations co-exist within a GBM, suggesting clonal diversity is an important factor for intratumoral heterogeneity. [2–6] On the other hand, glioblastoma stem/or initiating cell (GSC) model postulates cellular hierarchy with GSCs at the apex. These two models are non-mutually exclusive and can bring more comprehensive perspective to our understanding of GBM biology and therapeutics.

Although there are ongoing debates regarding GSC-defining surface marker, frequency, and reversibility of the cellular state, recent studies have suggested that GSCs are critical for GBM propagation and treatment resistance.[7–10] For instance, CD133-enriched GSCs contribute to radioresistance through the enhanced capacity of DNA damage repair.[11, 12] In addition, GSCs harbor high activation levels of the stem cell regulators and developmental pathways. These pathways include Sox2, WNT, Notch, and hedgehog signaling. Sox2 is a master regulator of stem cell maintenance in embryonic stem cells, tissue specific stem cells, and cancer stem-like cells. The WNT pathway is critical for self-renewal, proliferation, and differentiation of neural stem/progenitor cells and their progenies in the brain. We and others have shown the deregulation of WNT pathways in malignant brain tumor [13, 14] and that inhibition of the WNT signaling impedes tumor growth. Indeed, dozens of small molecule inhibitors that can inhibit WNT signaling have been developed for anti-cancer agents.

The forkhead box M1 (FoxM1) transcription factor plays critical roles in developmental processes and cancer by regulating the expression of cell cycle related genes, apoptosis, and DNA damage repair.[15–17] FoxM1 is a key mediator of aberrant WNT signaling in GBM via facilitating nuclear transport of β-catenin.[18] It also contributes to chemo-resistance by upregulation of the DNA damage repair signaling or MELK-mediated oncogenic signaling.[19] The role of FoxM1 in chemo-resistance of cancer has been further confirmed in multiple cancer types such as breast [20, 21], lung [22, 23], and colorectal cancer.[24] In contrast, much less is known for the role of FoxM1 in GBM radioresistance.

Several recent reports have suggested that FoxM1 might be more specifically associated with stem cell state in GBM.[13, 19] As GSC targeting is considered as a highly promising approach to treat GBM, FoxM1 inhibition can be an effective mean to target GSC-like cells. However, molecular links between FoxM1 and core stem cell regulator pathways remain incompletely understood. Based on this background, we investigated functional roles of FoxM1 in the core stem cell pathways and radioresistance. Utilizing primary patient-derived GBM cells and patient specimens, we showed that FoxM1 activates the stem cell pathways via transcriptional upregulation of Sox2, and this FoxM1-Sox2 signaling axis regulates radioresistance of GBM cells.

## Materials and Methods

### GBM patient-derived cells

All GBM specimens were collected with written informed consent under a protocol approved by the Institutional Review Board of the Samsung Medical Center (2010-04-004, Seoul, Korea). Following signed informed consent, tumor samples were obtained from GBM patients and dissociated into single cells. For in vitro expansion, patient-derived cells were cultured and passaged in Neurobasal A media (Invitrogen, Camarillo, CA) supplemented with B27 and N2 supplements (0.5X each; Invitrogen, Camarillo, CA) as well as recombinant EGF and bFGF (20 ng/ml each; R&D Systems, Minneapolis, MN).[25] To mimic differentiation of GBM cells, cells were starved in Neurobasal A media without any supplements for 1 day and then replaced with Neurobasal A media containing 5% (forced condition) or 0.1% (mild condition) FBS (GIBCO, USA), respectively.

### Orthotopic xenograft mouse model

Animal experiments were approved by the Institutional Review Boards of the Samsung Medical Center (20140312004, Seoul, Korea) and conducted in accordance with the “National Institutes of Health Guide for the Care and Use of Laboratory Aminals”. 5x10^4^ GBM cells resuspended in 5 μl Hank’s Buffered Salt Solution (GIBCO, USA) were stereotactically injected into the brain of 6-years old BALB/c-nu mice (n = 5–8 for each sample, weigh; 18±2 g). After 12 days from injection date, the brains of mice were irradiated with 2 Gy for 5 days (2 Gy x 5 times). Mice with the 20% body loss were sacrificed by CO_2_ asphyxiation. The brain tissue were fixed and stored as frozen tissue and FFPE for immunostaining. The survival of the mice was presented by Kaplan-Meier survival curves. P-value was calculated by Mantel-Cox test.

### Irradiation

GBM cells were irradiated as single cells using IBL 437C blood Irradiator (CIS US, Inc., Bedford, MA).

### Lentivirus production and virus infection

The shRNA knockdown plasmids targeting FoxM1 were purchased from Sigma Aldrich, USA. The shRNA knockdown plasmid sequence was as followed: pLKO.1-FoxM1 clone 1: CCGGGCCAATCGTTCTCTGACAGAACTCGAGTTCTGTCAGAGAACGATTGGCTTTTT. The FoxM1-overexpression plasmid was generated by PCR cloning of a FoxM1 open reading frame (ORF) sequence. The FoxM1 ORF was amplified from the plasmid from Origene (China) and its sequence was confirmed (NM_021953.3). And Sox2 coding sequence was amplified from patient-derived GBM cells and validated by sequencing. These fragments were subcloned into a pLenti6/V5-D-TOPO or pLenti6.3/V5-DEST plasmid (Invitrogen, Camarillo, CA). To generate recombinant lentivirus, these constructs were cotransfected with lentiviral packaging vectors (VSVG and PAX2) into HEK293FT cells (Invitrogen, Camarillo, CA) by using lipofectamine 2000 (Invitrogen, Camarillo, CA). Then, media supernatants were collected three times at intervals of 1 day, and lentiviruses were concentrated. For transduction of GBM cells, lentiviruses were added into the culture medium for 2 days. Puromycin (1 μg/ml) or blasticidin (2 μg/ml) selection was performed to remove non-infected GBM cells.

### Electroporation

The FoxM1 human cDNA ORF clone (Origene, China) was transfected into GBM cells using Neon^TM^ electroporation system according to the manufacturer's protocol (Invitrogen, Camarillo, CA). Briefly, the mixture containing 10^6^ GBM cells and plasmid was treated with double pulses of 1400 V for 20 ms in a 100 μl electroporation tip. Then, these cells were resuspended with Neurobasal A media without antibiotics.

### Immunohistochemistry and immunoreactivity score (IRS)

Glioma patient-derived clinical specimens were fixed by formalin and embedded in paraffin. Sections of paraffin-embedded glioma specimens were stained with a polyclonal antibody against human FoxM1 (MPP-2, K-19; Santa Cruz, USA) at a dilution of 1:50. To analyze immunohistochemical staining, at least 3 fields were randomly selected for each specimen. Quantitation of staining was performed by IRS system in which the percentages of staining positive cells and staining intensities were calculated.[26, 27] The percentages of positive cells were rated as follows: 1, less than 10% positive cells; 2, 11–50%; 3, 51–80%; 4, more than 80% positive cells. The classification of staining intensity was followed; 0, no color reaction; 1, mild reaction; 2, moderate reaction; 3, intense reaction. The sections stained with anti-FoxM1 antibody were classified by IRS as followed: 0–1; negative; 2–3; mild, 4–12; strong staining.

### Nuclear/cytoplasmic fractionation

Nuclear/cytoplasmic lysates were isolated using NE-PER Nuclear/Cytoplasmic Extraction Reagents by following the manufacturer's protocol (Thermo Fisher Scientific, USA). Each lysate was used for western blotting analysis and we requisitely detected these antibodies; FoxM1 (Santa cruz, USA), Sox2 (R&D Systems, Minneapolis, MN), TATA-box binding protein (TBP, Cell signaling, USA) and α-tubulin (Abclone, Korea).

### RNA isolation and real-time quantitative PCR

Total RNA was isolated from GBM cells using an RNeasy Mini Kit (Qiagen, USA). Then, reverse transcription was conducted using a SuperScript^TM^ III First-strand cDNA synthesis kit (Invitrogen, USA). The relative amount of mRNA was evaluated using Applied Biosystems 7500 Real-Time PCR System (Applied Biosystems, USA). The primer sequences were followed: 5’-TGCCCAGCAGTCTCTTACCT-3’ (forward) and 5’-CTACCCACCTTCTGGCAGTC-3’ (reverse) for human FoxM1, 5’-TGCTGCCTCTTTAAGACTAGG-3’ (forward) and 5’-CCTGGGGCTCAAACTTCTCT-3’ (reverse) for human Sox2, 5’- GAGGCACTCTTCCAGCCTTC-3’ (forward) and 5’- GGATGTCCACGTCACACTTC-3 (reverse) for humanβ-acin used as an internal standard for normalization.

### Western blot

Protein lysates were prepared in NP-40 lysis buffer (Invitrogen, Camarillo, CA) consisted of 1% NP-40, 5% glycerol, 20 nmol/L NaF, 5 mmol/L EDTA, 5 mmol/L EGTA, and freshly added protease inhibitor cocktail and 1 mM PMSF. The membrane was incubated with antibodies against interesting protein at 4°C overnight. The signals from the primary antibodies were amplified by HRP-conjugated secondary antibodies and detected with ECL solution (Amersham Biosciences, USA). The following antibodies were used: anti-FoxM1 (santa cruz, USA); anti-α-tubulin (Abclon, Korea); anti-TBP (CST, USA); anti-Bax and anti-PARP (Cell signaling, USA).

### Limiting dilution assay

Tumor cells from the patient GBM cells or xenograft tumors derived from GBM patient cells were enzymatically dissociated into single cells, and then plated into 24 wells of 96 well plates with different densities (1–500 cells per well). Cells were incubated at 37 C for 2 weeks. At the time of quantification, each well was examined for the formation of neurospheres. For a statistical analysis, the numbers of responded events were plotted and stem cell frequency was calculated using the Extreme Limiting Dilution Analysis software.

### Chromatin immunoprecipitation

Cells were cultured under the medium with DMSO or 2 μM Siomycin A for a day and crosslinked with 1% formaldehyde. The cells were lysed and sonicated on ice. Anti-FoxM1 antibody or IgG were added to chromatin lysates, and incubated overnight at 4 C. Then we subjected the DNA to amplify the FoxM1 binding sites on Sox2 promoter and analyzed.

### Immunocytochemistry

Chamber slides were pre-coated with poly-L-ornithine (Sigma Aldrich, USA) and 2x10^4^ single cells were seeded per well 4 hours before the actual experiments. To staining these cells, the cells were fixed with 4% paraformaldehyde (PFA) and incubated at room temperature. Then blocking solution (5% NHS and 0.5% Triton X-100 in PBS) was added at room temperature for 1 hour. And then the cells were stained with antibodies diluted in antibody dilution buffer (1% BSA and 0.3% Triton X-100 in PBS). The following antibodies were used as primary antibodies: anti-FoxM1 (Santa cruz, USA); anti-Sox2 (R&D Systems, Minneapolis, MN); anti-Nestin conjugated to Alexa-647 (BD Biosciences, San Jose, CA, USA); anti-pH2AX (Millipore, Billerica, MA); anti-GFAP (Millipore, Billerica, MA); anti-O4 (Millipore, Billerica, MA); anti-Tuj1 (Millipore, Billerica, MA), anti-NeuN (R&D Systems, Minneapolis, MN). Analysis was performed using confocal LSM 700 (Carl Zeiss, Germany).

### TUNEL assay

TdT-mediated dUTP Nick End Labeling (TUNEL) assay was performed using ApopTag Fluorescein *In Situ* Apoptosis Detection Kit (Intergen Company, USA). After the cells were exposed to radiation, they were incubated on chamber slide for 4 days. Then they were fixed with 4% PFA, washed in equilibration buffer, and incubated with TdT enzyme in a humidified chamber at 37 C for 1 hour. Afterwards, they were washed and incubated at room temperature for 30min in the dark chamber with fluorescein-conjugated anti-digoxigenin. Washed cells were counterstained with DAPI. The stained cells were analyzed using confocal LSM 700 (Carl Zeiss, Germany).

### Flow cytometry and cell cycle analysis

Patient-derived GBM cells were dissociated into single cells, and then permeabilized using the BD Cytofix/Cytoperm^TM^ buffers (BD Biosciences, San Jose, CA, USA). The cells were labeled with the following antibodies: anti-FoxM1 (Abcam, UK), anti-Sox2 (Cell signaling, USA). To analyze the cell cycle, cells were fixed with 100% ethanol and incubated at 4 C for a day. The cell cycle disposition of cells stained with PI (Sigma Aldrich, USA) was analyzed using Flow Cytometry (FASC Calibur, BD Biosciences, San Jose, CA, USA), and the flow cytometry data were analyzed with a Flow Jo^TM^ software (Treestar Inc, USA)

## Results

**Fig 1:**
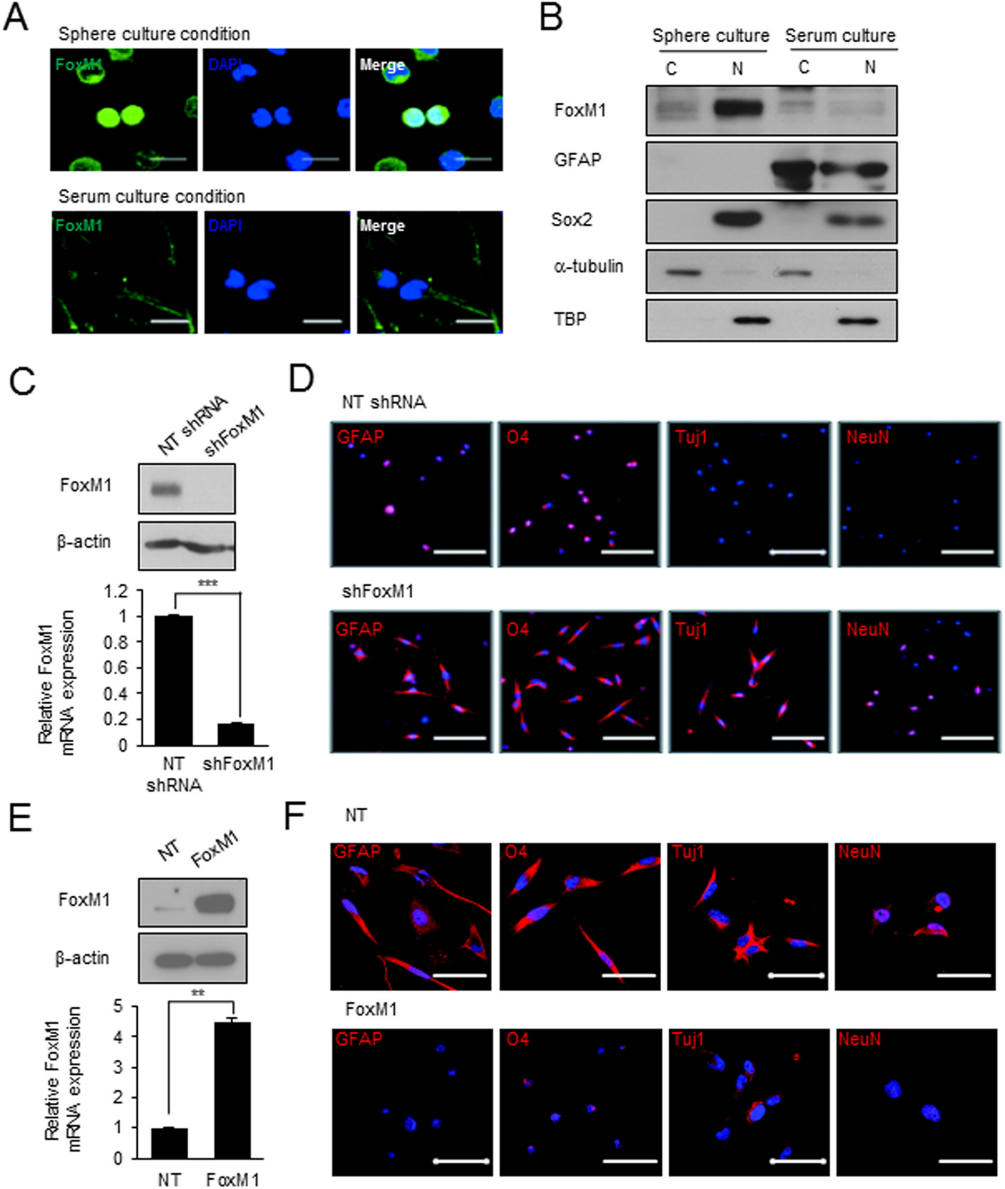
FoxM1 knockdown increases, and overexpression decreases, differentiation-associated marker expression in primary GBM cells. (A) Representative immunofluorescence images stained with anti-FoxM1 antibody in NS07-448 cells that were cultured under sphere culture condition or 5% serum culture condition. Scale bar = 20 μm. (B) Western blotting analysis of FoxM1, Sox2, and GFAP (astroglial differentiation marker) using nuclear (N) or cytoplasmic (C) protein lysates from sphere cultures and serum cultures. As loading controls, α-tubulin (cytoplasm) and TBP (TATA-box binding protein, nuclear) were used. (C) FoxM1 expression in FoxM1-knockdown cells. β-actin was used as a loading control. (D) Differentiation marker expression in the control and FoxM1-shRNA expressing cells under differentiation-inducing conditions (0.1% serum culture condition for 7 days). Cells were stained with antibodies for neural cell differentiation markers (GFAP, astroglial marker; O4, oligoglial marker; TuJ1 and NeuN, neuronal marker). Scale bar denotes 100 μm. (E) FoxM1 expression in FoxM1-overexpressing cells. (F) Differentiation marker expression in the control and FoxM1-overexpressing cells under differentiation-inducing conditions. Ectopic expression of FoxM1 was achieved by electroporation with a FoxM1 expression vector. The cells were stained with the same markers used in (D). Scale bar, 50 μm.

We and others previously showed that stem associated characteristics of primary GBM cells are well maintained when these cells were cultured in serum-free culture conditions supplemented with EGF and FGF, compared to high serum-contained media condition.[8, 9] In spheroid culture conditions, we found that primary GBM cells have high expression levels of various GSC enrichment markers such as CD133, CD15, c-Met, and Sox2, and these tumor cells harbor the capacities of clonogenic growth and tumor propagation.[8, 9] To check differentiation status, the cells were cultured under serum contained media and stained with differentiation markers. Expression of the differentiation markers, such as GFAP, O4, Tuj1 and NeuN (astrocyte, oligodendrocyte and neuron, respectively) exhibited with increasing tendency (S1 Fig). To investigate the potential association between FoxM1 expression and stem cell status, we determined FoxM1 expression in GBM cells that were cultured in sphere and serum culture condition (Fig 1). Whereas FoxM1 proteins are highly expressed in the GBM cells in sphere culture condition, FoxM1 expression was hardly detected in the GBM cells in the serum culture condition (Fig 1A). As FoxM1 is a transcriptional factor, nuclear localization of FoxM1 is important for its function. We determined subcellular localization of FoxM1 in GBM cells in these two culture conditions (Fig 1B). Consistent with immunofluorescence data, robust expression of FoxM1 was detected only in spheroid-cultured GBM cells. These data together provide an initial link between the FoxM1 and stem cell state.

Next, to determine whether FoxM1 plays active roles in GBM differentiation state, we inhibited or overexpressed FoxM1 in GBM cells and evaluated their effects on differentiation marker expression. By transduction with FoxM1 shRNA-expressing lentiviru, we decreased FoxM1 protein levels by about 80% of the control (Fig 1C). Compared to the control cells, FoxM1 shRNA-expressing GBM cells revealed high expression of differentiation markers including GFAP (astroglial) and TuJ1 (neuronal) (Fig 1D). Conversely, FoxM1-overexpressing GBM cells had much lower expression levels of differentiation-associated markers compared to the control cells, albeit these cells were cultured in differentiation-prone conditions (Fig 1E and 1F). Taken together these data indicate that FoxM1 may actively control the differentiation states of primary GBM cells.

### Association between Sox2 and FoxM1 signaling in GBM

**Fig 2:**
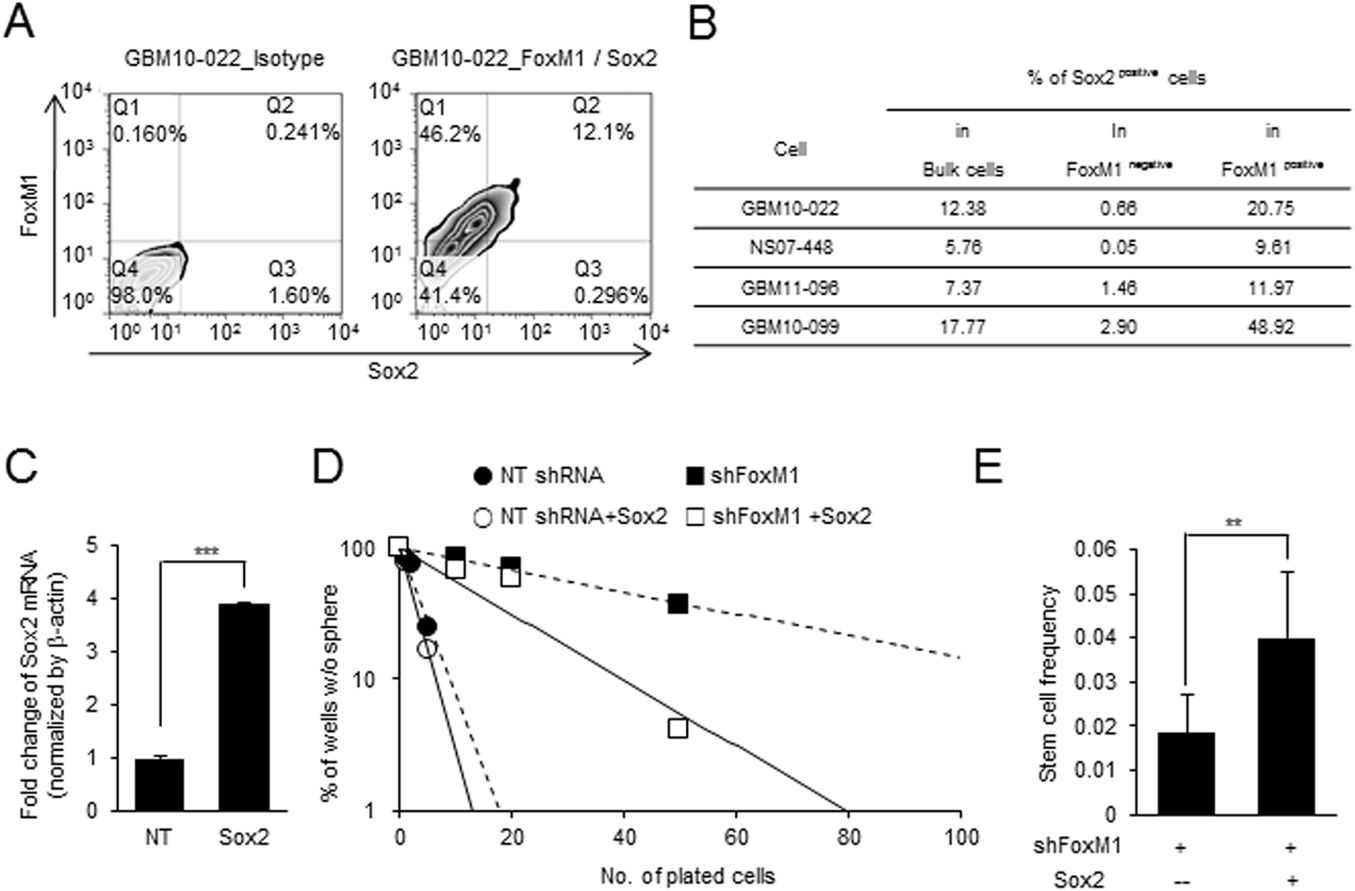
FoxM1 knockdown decreases and Sox2 overexpression partially rescues clonogenic growth of GBM cells. (A) FACS analysis of GBM cells to determine co-localization of FoxM1 and Sox2. (B) Four different GBM cells stained with anti-FoxM1 and anti-Sox2 antibodies were analyzed by flow cytometry. (C) Sox2 mRNA expression in Sox2-overexpressing cells. GBM11-096 GBM cells were transduced with lentivirus either expressing the mock control (NT) or Sox2. Expression levels of Sox2 mRNA were determined by real-time PCR. (D) Sphere formation limiting dilution assays using GBM cells with FoxM1 knockdown, Sox2 overexpression, or both. (E) Estimated frequencies of clonogenic cells in each group were determined by ELDA analysis.

Primary GBM cells are the mixture of stem-like cells and more differentiated tumor cells. Utilizing four different primary GBM cells, we determined FoxM1 and Sox2 expression by flow cytometric analysis (Fig 2A and 2B). Tumor cells expressing high levels of Sox2 represent undifferentiated tumor cells. Consistent with in vitro data, FoxM1-expressing cells were highly enriched in Sox2 positive tumor cells compared to Sox2 negative/low tumor cells. Additionally, we checked Sox2 expression in FoxM1 knockdown cells (S2 Fig). Sox2 in FoxM1 knockdown cells was significantly decreased to about 50% in the control cells.

As a potential mechanism of the FoxM1 signaling in GBM stem cell maintenance, we hypothesized that FoxM1 may transcriptionally regulate key stem cell regulators. To determine whether Sox2 is functionally important for the FoxM1 signaling, we determined the clonogenic growth of GBM cells with the modulation of these two proteins. Previous studies have shown that FoxM1 knockdown decreased the clonogenic growth.[28] Consistent with this, FoxM1 knockdown GBM cells impaired cell growth and decreased the frequencies of clonogenic cells. Importantly, Sox2 overexpression in FoxM1 knockdown cells significantly rescued cell growth (Fig 2C–2E). These data, together, are consistent with the hypothesis that Sox2 is a downstream target of FoxM1 signaling in GBM.

### Sox2 is transcriptionally regulated by FoxM1 in GBM

**Fig 3:**
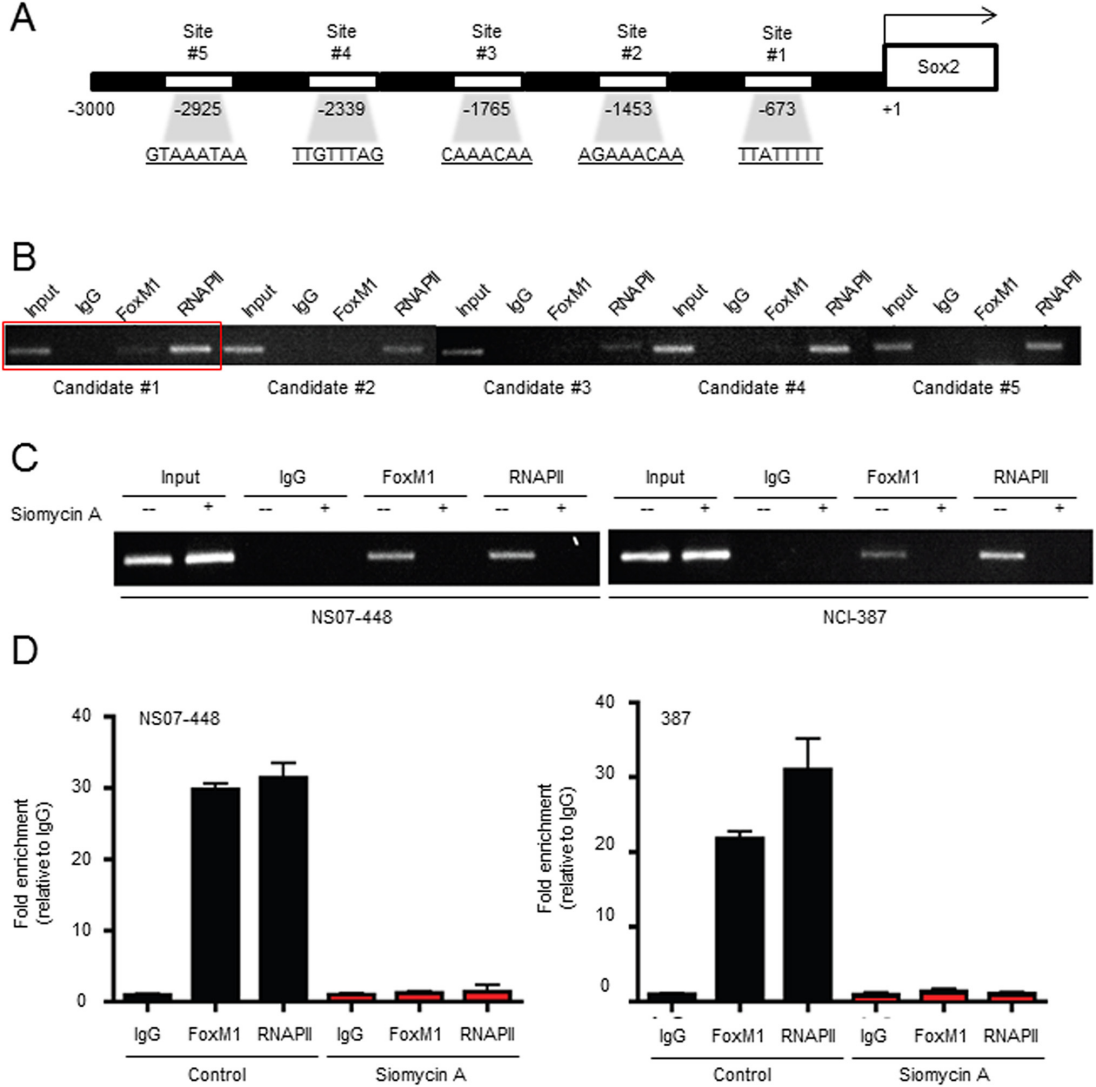
FoxM1 binds to Sox2 promoter region and regulates its expression. (A) Diagram of potential FoxM1-binding sites on Sox*2* promoter region. RNAPII was used as a positive control. (B) CHIP-PCR analysis to determine the FoxM1 binding site on Sox2 promoter. Lysates of NS07-448 cells were incubated with IgG, FoxM1 or RNAP II (RNA polymerase II) and processed for CHIP-PCR. (C) CHIP-PCR analysis using NS07-448 and 387 cells treated with a small-molecule FoxM1 inhibitor Siomycin A (2 μM) for 24 hours. (D) Quantitation of PCR bands shown in (C).

To demonstrate FoxM1-mediated transcriptional upregulation of Sox2 in GBM, we analyzed genomic sequences of Sox2 promoter regions. FoxM1 protein includes 3 domains; a N-terminal repressor domain, a C-terminal transactivation domain and a conserved forkhead DNA binding domain.[29, 30] Potential binding sites for FoxM1 transcription factor include the consensus binding site [A(T/C)AAA(T/C)AA] or consensus FOXO recognition elements (FHRE) [(G/C)(T/C)AAA(T/C)AA or TT(G/A)TTT(G/A)(G/C)]. Examination of Sox2 promoter revealed 5 candidate sites within 3 kb region of the human Sox2 gene (Fig 3A). By chromatin immunoprecipitation-PCR analysis, we found that FoxM1 proteins immunoprecipitated with candidate #1 DNA region among the 5 candidate sites (Fig 3B). To further validate this finding, we inhibited FoxM1 by a small-molecule inhibitor Siomycin A and performed similar CHIP-PCR analysis. Binding of FoxM1 to the Sox2 promoter region was near completely inhibited by Sinomycin A (Fig 3C and 3D). Together, these data support that FoxM1 transcriptionally regulates Sox2 in GBM cells.

### FoxM1 and Sox2 proteins are upregulated in the surviving GBM cells after irradiation

**Fig 4:**
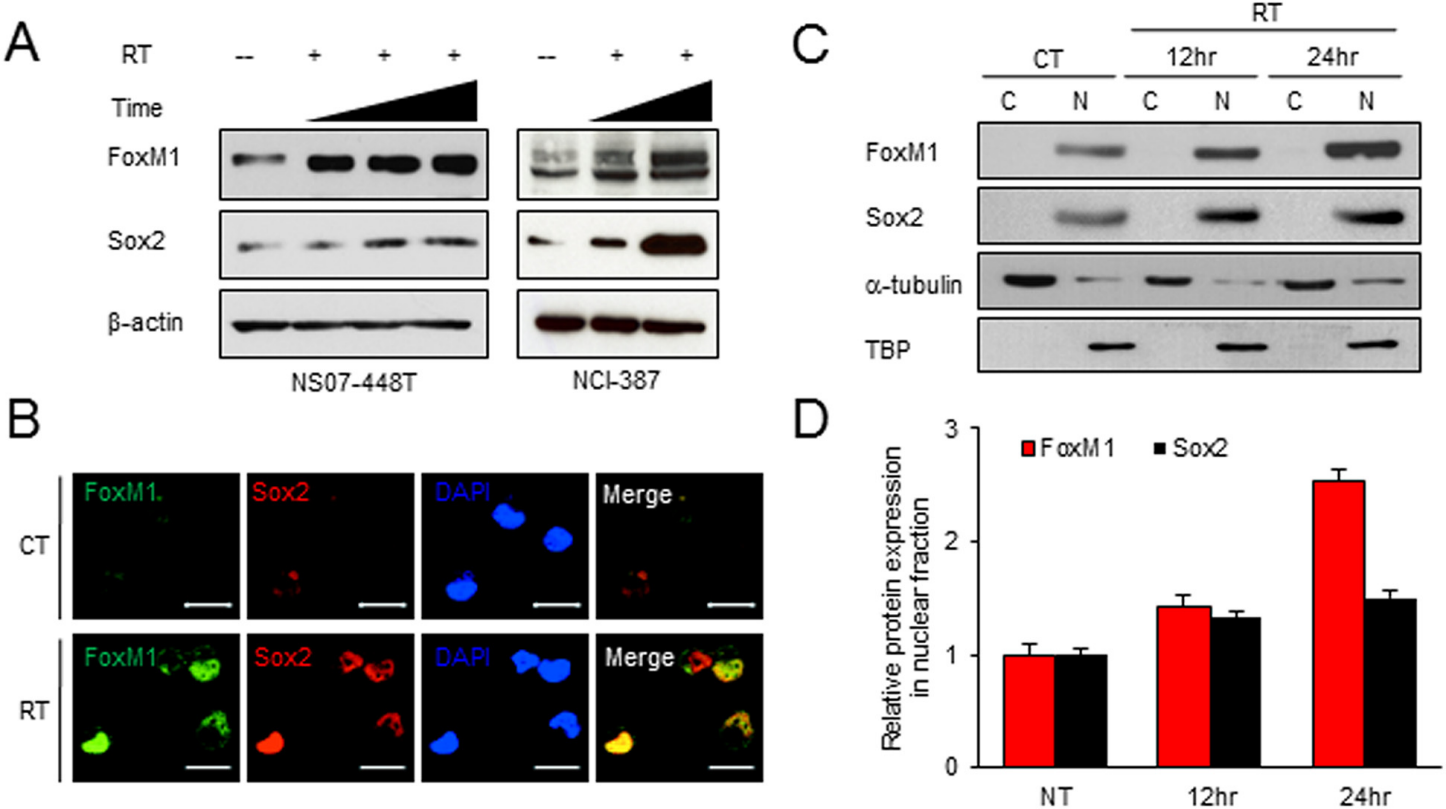
FoxM1 and Sox2 are upregulated in GBM cells after irradiation. (A) Western blot analysis of FoxM1 and Sox2 using the irradiated NS07-448 (left) and 387 (right) GBM cells. Cells were harvested with course of time after irradiation. β-actin was used as a loading control. (B) Representative IF images of NS07-448 GBM cells with or without irradiation using anti-FoxM1 and anti-Sox2 antibodies. Scale bar, 20 μm. (C and D) Western blot analysis of FoxM1 and Sox2 in GBM cells after irradiation. Cytoplasmic (C) and nuclear (N) fractions of protein lysates were prepared to determine FoxM1 and Sox2 changes 6 and 12 hours after irradiation. As loading controls, α-tubulin (cytoplasm) and TBP (nuclear) were used. Quantitation of these protein bands were shown in (D).

Radiation treatment remains the most effective therapy for GBM patients except surgery. Unfortunately, GBM cells almost always develop resistance mechanisms. Overcoming these radioresistance mechanisms may significant benefit to the patients. To determine whether FoxM1 signaling is involved in GBM radioresistance, we irradiated GBM cells and determined protein levels in the surviving GBM cells after irradiation (Fig 4A). FoxM1 levels were strikingly increased in surviving GBM cells. Interestingly, Sox2 protein levels were also increased with a slightly delayed induction pattern. We then determined upregulation of these proteins by IF analysis (Fig 4B). Compared to the untreated control cells, surviving tumor cells after irradiation revealed high expression of both FoxM1 and Sox2. To further determine induction kinetics of FoxM1 and Sox2 after irradiation, we harvested cells 6 or 12 hours after irradiation and performed Western blot analysis. Both FoxM1 and Sox2 levels were increased over time, but FoxM1 accumulation preceded the Sox2 accumulation (Fig 4C).

### FoxM1 knockdown sensitizes GBM cells to radiation

**Fig 5:**
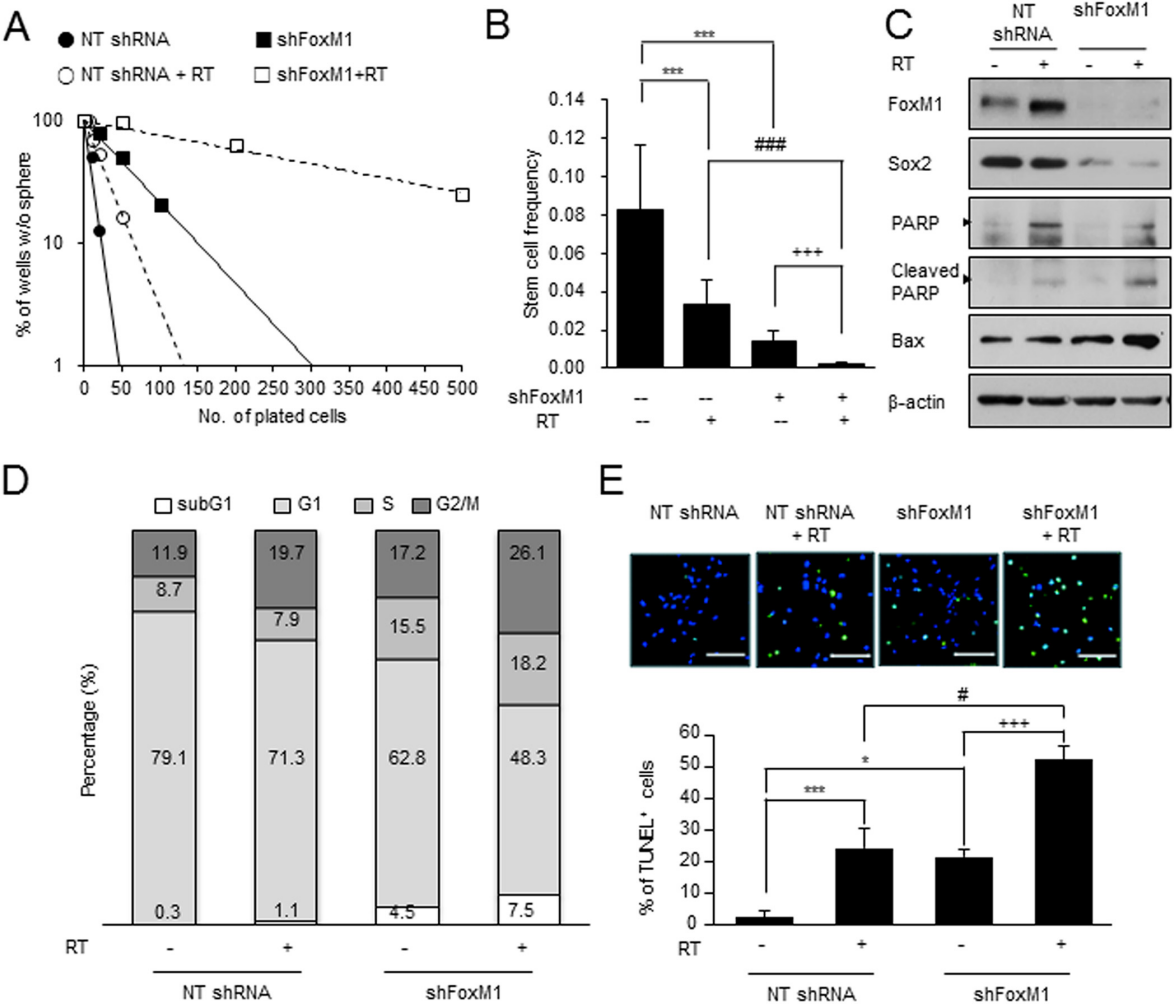
FoxM1 knockdown sensitizes GBM cells to irradiation. (A) LDA analysis of the control and FoxM1 knockdown NS07-448 GBM cells with or without *in vitro* irradiation. (B) Change in the clonogenicity of GBM cells by FoxM1 knockdown and/or *in vitro* irradiation was evaluated by the limiting dilution assay. Sphere frequencies were calculated using ELDA. (C) Western blot analysis of FoxM1, Sox2 and apoptosis markers (cleaved PARP and Bax) in GBM cells with FoxM1 knockdown and irradiation treatments. β-actin was used as a loading control. (D) Cell cycle analysis of GBM cells with FoxM1 knockdown, irradiation, or both. Cell cycle distribution was determined by flow cytometry. P-values were calculated using the Fisher’s exact test (p<0.001). (E) Representative TUNEL assay images from the cell groups (top). Scale bar, 100 μm. Quantitation of TUNEL-positive apoptotic cells in each group (bottom). *** and *, p<0.001 and <0.05 compared to NT shRNA, respectively; #, p<0.05 compared to NT shRNA+RT; +++, p<0.001 compared to shFoxM1.

To evaluate functional significance of FoxM1 in radiation resistance of GBM, we inhibited FoxM1 by shRNA-mediated knockdown and assessed its effects on tumor cell survival and growth. First, we determined clonogenic growth of NS07-448 GBM cells by limiting dilution clonogenic assays. FoxM1 knockdown or irradiation alone decreased the clonogenic capacities of these cells by 2.5 fold and 5.8 fold, respectively. Notably, combination of FoxM1 knockdown and irradiation almost completely inhibited clonogenic growth of these cells, suggesting a synergistic effect (Fig 5A and 5B). As we found upregulation of FoxM1 and Sox2 in the surviving GBM cells after irradiation, we determined Sox2 levels in the FoxM1 knockdown cells with or without irradiation. Sox2 expression was significantly decreased in FoxM1 knockdown cells after irradiation (Fig 5C).

Diminished GBM clonogenic growth by combination of FoxM1 inhibition and irradiation prompted us to examine the effects on cell cycle kinetics and survival. Irradiation or FoxM1 knockdown alone induced cell cycle arrest in G2/M phase and subsequent apoptosis of GBM cells. In concordance with the clonogenicity data, FoxM1 knockdown combined with irradiation resulted in the significant increase in the G2/M and subG1 cell population compared to other groups (Fig 5D). TUNEL analysis and western blot analysis also indicated significantly increased degree of apoptosis in the FoxM1 knockdown and irradiation group (Fig 5B and 5E). Taken together, these data support the notion that FoxM1 is a key regulator for GBM radioresistance.

### FoxM1 knockdown combined with irradiation impeded tumor growth in orthotopic GBM xenograft tumor models

**Fig 6:**
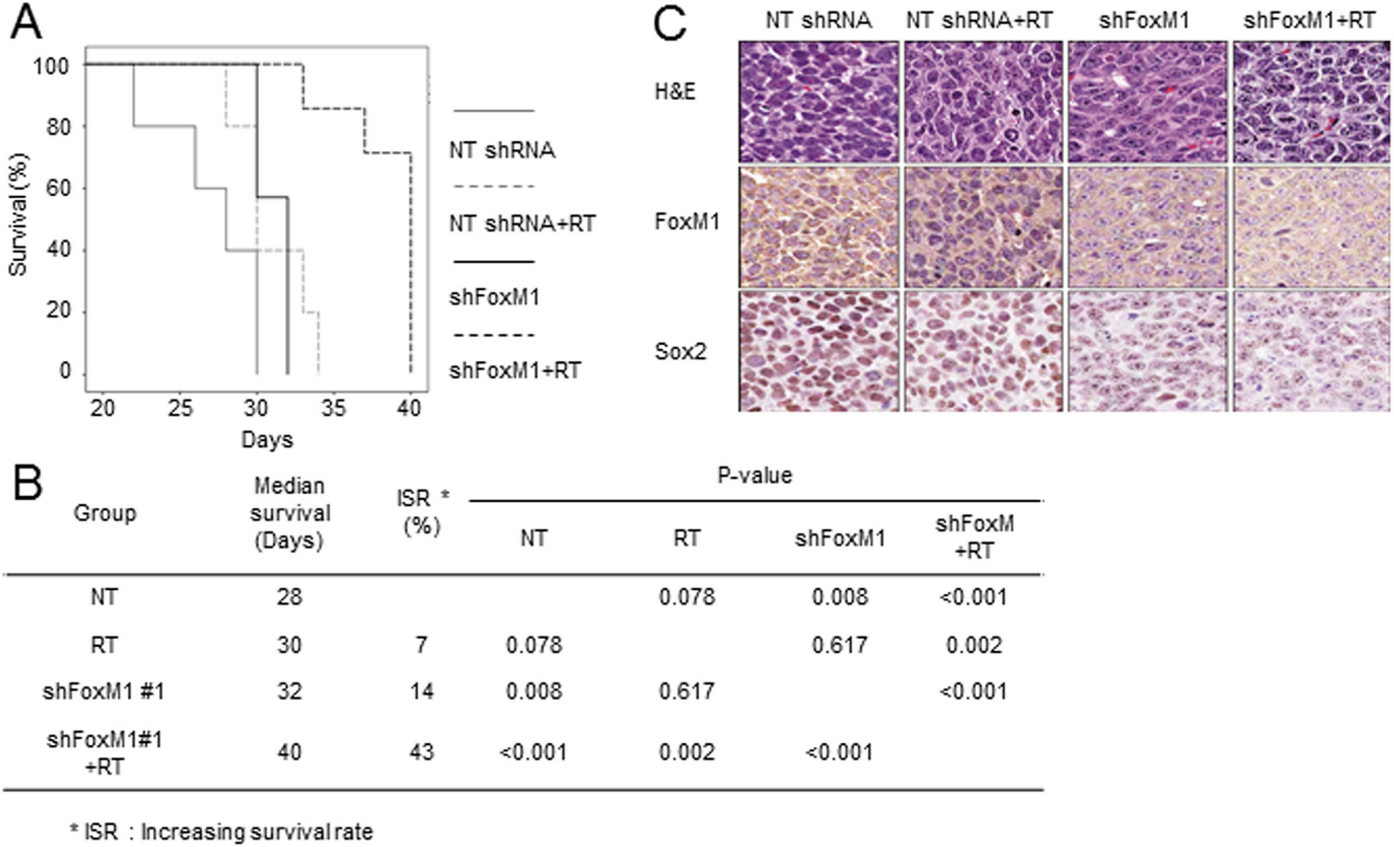
FoxM1 knockdown combined with *in vivo* irradiation significantly prolonged the survival of tumor bearing mice. (A) Kaplan-Meier survival curves of orthotopic tumor bearing mice. Control or FoxM1 expressing 387 GBM cells were intracranially injected into the brain of mice. Two weeks later tumor implantation, *in vivo* irradiation directed to the brains was performed (2 Gy daily does for 5 days). (B) Summary of survival and statistical significance. (C) Representative immunohistochemical images using FoxM1 and Sox2 antibodies on xenograft tumor sections. The brain sections were paraffin-embedded and stained with anti-FoxM1 and anti-Sox2 antibodies. H&E staining was used to visualize tissue histology.

The above *in vitro* data collectively suggest that FoxM1 targeting can be an effective anti-GBM therapy, especially in combination with irradiation. To test this in a pre-clinical animal model, we have set up *in vivo* tumor models. Control or FoxM1 knockdown GBM cells were injected into the brains of immune-deficient nude mice. Once tumors were formed, fractionated irradiation was applied to these mice. Animal survival, tumor histology, and immunostaining analysis were evaluated in these mice (Fig 6). Compared to control group (median survival = 28 days), irradiation only group or FoxM1 knockdown only group (shFoxM1 #1) exhibited slightly longer median survival by 7% (median survival = 30 days, p-value = 0.78) or 14% (median survival = 32days, p-value = 0.008), respectively (Fig 6A and 6B). Importantly, mice received the combined treatment showed a significantly prolonged median survival (median survival = 40 days, p<0.005). We further analyzed FoxM1 and Sox2 expression in brain tissues obtained from the xenograft mice (Fig 6C). FoxM1 knockdown combined with irradiation group exhibited very low levels of Sox2 compared to control and sole irradiation groups. Together, these *in vivo* results strongly support the efficacy of FoxM1 knockdown combined with irradiation for anti-GBM therapy.

### FoxM1 expression correlates with glioma grades and portends poor patient survival

FoxM1 expression in glioma patients has been documented in the previous reports, but the number of specimens examined was rather small or only FoxM1 mRNA expression was evaluated in some cases. To more comprehensively investigate the expression levels of FoxM1 in clinical specimens and potential association between its expression and clinical parameters, we have analyzed the data from multiple GBM databases. First, examination of the Rembrandt glioma database showed that FoxM1 expression in gliomas was significantly higher than that in normal brain tissues and exhibited increasing tendency with the WHO grade of gliomas (grade II, III, and IV) (p-value<0.001, S3A Fig).

**Fig 7:**
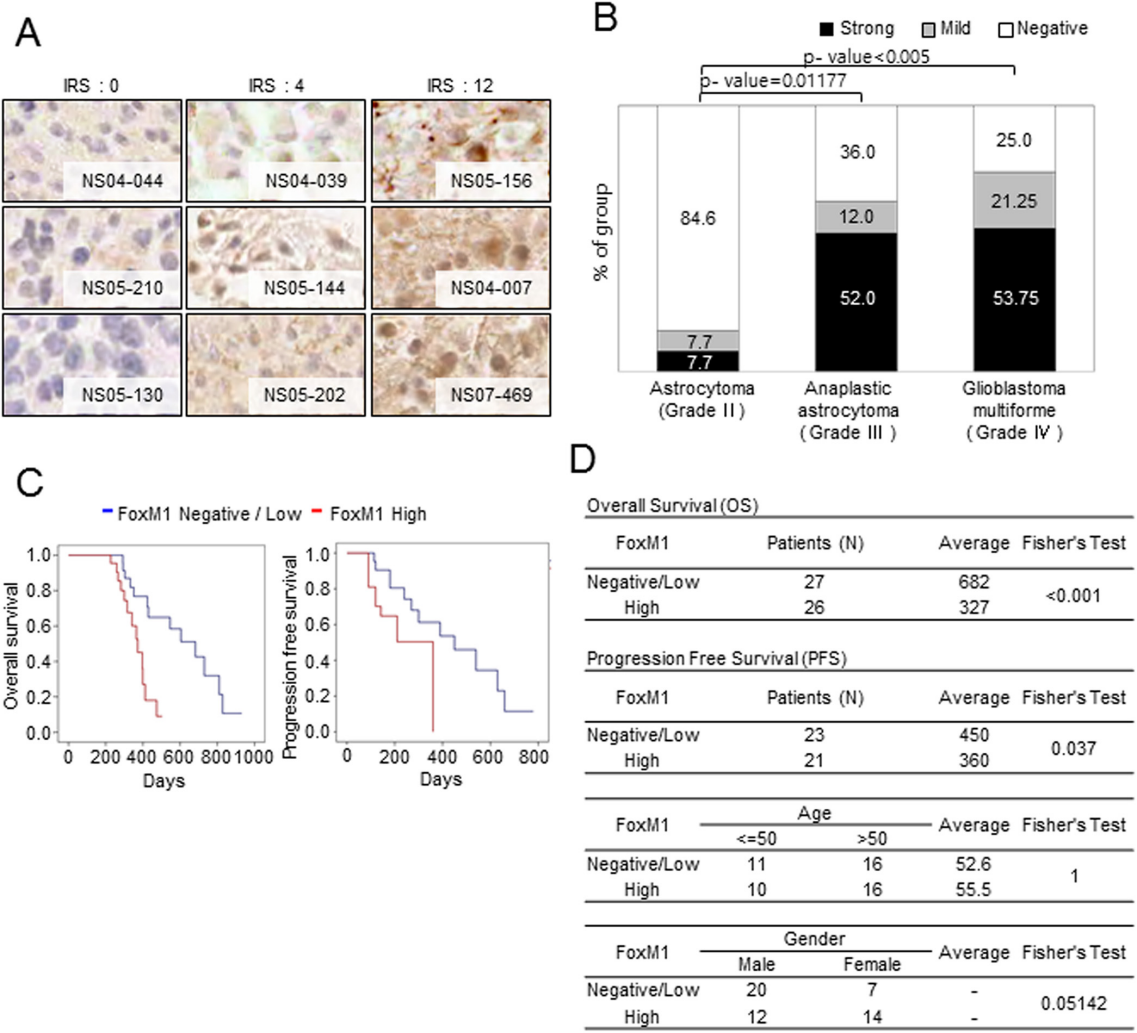
FoxM1 expression levels in Clinical glioma specimens and association with patient survival. (A) Representative images of FoxM1 staining analysis using tissue microarray (TMA) sections. FoxM1 protein level of each glioma section was quantified according to the IRS method. (B) FoxM1 protein levels of each WHO glioma grade were compared using the IRS score (High = 4–12, Mild = 1–3, Low = 0). P-values were calculated using the Fisher’s exact test. (C and D) Kaplan-Meier survival curves and statistical analysis of glioma patients that were categorized based on the level of FoxM1. FoxM1 negative/low (blue) and high (red) groups were plotted. Overall survival (OS, left) and Progression-free survival (PFS, right) were compared by the Log rank test.

To verify these observations at the protein level, we determined FoxM1 expression using a tissue microarray (TMA) constructed in our laboratory, which harbors 13 astrocytomas (grade II), 25 anaplastic astrocytomas (grade III) and 80 GBM (grade IV) specimens. FoxM1 was detected both in the cytoplasm and nucleus of tumor cells with more prominent expression in nucleus, as previously reported.[27] To quantify FoxM1 protein level, we have applied an immunoreactive score (IRS) method (Fig 7A).[26, 31] FoxM1 protein level was rarely detected in low-grade glioma patients (15.4% of grade II was positive), whereas grade III and grade IV glioma specimens exhibited significantly higher FoxM1 level (64% of grade III and 75% of grade IV were positive) (Fig 7B). These data indicate that FoxM1 protein expression levels positively correlate with the WHO grade of gliomas, further confirming the association between FoxM1 expression and glioma malignancy.

Next, we determined whether FoxM1 protein level is associated with clinical outcomes of GBM patients (Fig 7C and 7D). 53 GBM specimens among 80 GBM specimens in the TMA were selected because these specimens are supplemented with clinical information (S1 Table). These specimens were classified into two groups based on the IRS score of FoxM1 expression [negative/low (IRS = 0–3, n = 27) vs. high group (IRS = 4–12, n = 26)]. As shown in Fig 7C (left), OS of FoxM1 negative/low GBM patients (Median = 682 days, Range = 292~931 days) was significantly longer than that of FoxM1 high GBM patients (Median = 372 days, Range = 227~504 days, p-value<0.001). FoxM1 negative/low GBM patients also showed significantly longer PFS (Median = 450 days, Range = 114~780 days, p-value = 0.037, Fig 7C, right) than FoxM1 high GBM patients (Median = 360 days, Range = 114~360 days). `

Similarly, analysis of the TCGA datasets revealed that a subset of GBM patients with high FoxM1 mRNA expression (high 10%, n = 46) showed poor overall survival than the patients with low FoxM1 (low 10%, n = 46), although it was not statistically significant (S3B and S3C Fig, p-value = 0.086). These data suggest that FoxM1 can be a prognostic marker for glioma malignancy.

## Discussion

In this study, we showed that FoxM1 maintains GBM stem cell state in part via FoxM1-mediated transcriptional regulation of the key stem cell regulator Sox2, and that the FoxM1-Sox2 signaling axis is activated in the surviving GBM cells after irradiation. We further showed that FoxM1 targeting either by shRNA-mediated genetic inhibition or pharmacological inhibition significantly sensitized GBM cells to radiation. Therapeutic efficacy of the FoxM1 targeting combined with irradiation in a pre-clinical animal model suggests that FoxM1 is a promising therapeutic target for anti-GBM therapy.

FoxM1 signaling has been implicated in various oncogenic processes, including cell cycle, invasion, proliferation, angiogenesis, and DNA damage responses, to name a few. For example, FoxM1 potentiates WNT pathway activation by facilitating β-catenin nuclear accumulation and direct transcriptional upregulation of WNT pathway target genes.[13, 32] Other reports have shown that FoxM1 binds to VEGF promoter and regulates VEGF expression in glioma cells.[33, 34] In pancreatic cancer, FoxM1 stimulates epithelial-mesenchymal transition (EMT), invasion and angiogenesis.[35] In breast cancer, FoxM1 promotes EMT by regulating Slug expression and its knockdown inhibits mesenchymal phenotype.[36] Here, we showed that FoxM1 induces transcription of Sox2 in GBM cells. A direct binding of FoxM1 in the Sox2 promoter region in GBM cells was confirmed by CHIP-PCR analysis. Our finding is consistent with a recent paper that reported FoxM1-mediated Sox2 induction in neuroblastoma cells.[37] Importantly, our data that FoxM1 overexpression upregulated Sox2 expression in GBM cells, and that ectopic expression of Sox2 rescued FoxM1 inhibition-mediated effects further support that Sox2 is a key downstream regulator of FoxM1 signaling in GBM.

Sox2 is a well-defined key regulator of self-renewal capacity in both normal and neoplastic stem cells.[38–40] According to the cancer genome atlas (TCGA) data analysis, Sox2 is overexpressed in 86.2% (357/414) of GBM compared to the normal brain.[41] Amplification of Sox2 gene and hypomethylation of Sox2 promoter were observed in 8.5% (42/492) and 100% GBM specimens, respectively [41]. In addition, a recent report showed that overexpression of Sox2, along with other transcriptional factors POU3F2, SALL2, and OLIG2, was sufficient to acquire GSC-like properties.[42] Given the prominent roles of Sox2 in GBM malignancy, it is conceivable that FoxM1-Sox2 signaling axis may be a critical regulator of stem cell maintenance. In this context, FoxM1 targeting can be an indirect mean to inhibit Sox2 signaling. Alternatively, FoxM1 inhibition may inhibit multiple downstream signaling pathways. Intriguingly, cancer stem cell-like cells are known to express high levels of all the forementioned FoxM1 downstream effectors including WNT, EMT, and Sox2.

Chemo- or radiation-resistance acquired by tumor cells is a major reason to limit therapeutic benefit in cancer patients. The role of FoxM1 in chemoresistance has been reported in various cancers including GBM, but relatively little is known for radioresistance. *Huang* group and *Nakano* group recently reported that FoxM1 knockdown sensitized GBM cells to TMZ chemotherapy mainly through Rad51 downregulation [18] and interference of MELK-mediated signaling, respectively. In this study, we showed that FoxM1 knockdown sensitizes GBM cells to radiation both *in vitro* and *in vivo*. In contrast to the previous report, FoxM1 inhibition by shRNA-mediated knockdown provided rather subtle survival gain in tumor bearing mice in our study. Experimental factors such as FoxM1 knockdown efficacy, the usage of different tumor cells, and the number of injected tumor cells are likely reasons of this discrepancy. However, FoxM1 inhibition combined with irradiation significantly impeded tumor growth and prolonged survival of tumor bearing mice. We showed that FoxM1 expression is significantly increased in the surviving tumor cells after irradiation, consistent with a previous report in which FoxM1 expression was higher in recurrent GBMs compared to the primary GBMs.[43] Together, these findings further corroborate the rationale of FoxM1 targeting in combination with irradiation.

In summary, our data demonstrate that FoxM1 is a key regulator of clonogenic growth, stem-like properties, and radioresistance of GBM. FoxM1 targeting downregulated Sox2 expression and sensitized GBM cells to radiation both *in vitro* and *in vivo* animal model. High expression levels of FoxM1 are correlated with glioma malignancy and poor patient survival, and most GBM patients express high levels of FoxM1. Together, our findings suggest that FoxM1 targeting combined with irradiation is a potentially effective therapeutic approach for GBM.

## Supporting Information

S1 ARRIVE Checklist The ARRIVE Guidelines Checklist. (PDF)

S1 Fig Differentiation and neural cell marker expression of GBM cells. NS07-448 cells were cultured under two different culture conditions (sphere and serum culture condition). (A) The GBM cells were forced differentiation in serum culture condition (5% FBS) and stained with anti-neural differentiation markers antibodies (anti-GFAP, O4, Tuj1, and NeuN) to check differentiation status of GBM cells. Scale bar = 50 μm. (B) Signal intensities were analyzed and compared.
(PDF)

S2 Fig Sox2 expression in FoxM1 knockdown cells. GBM11-096 GBM cells were infected with lentivirus carring FoxM1 shRNA. FoxM1 and Sox2 expressions were presented; *** and **, p<0.001 and <0.005 compared to NT shRNA, respectively.
(PDF)

S3 Fig Clinical implications of FoxM1expression in glioma patients. FoxM1 mRNA expression in gliomas were derived from the public by Rembrandt database, which harbors 21 normal brain, 92 low grade gliomas, 69 anaplastic astrocytomas, and 126 GBM specimens, respectively. (B) GBM patients from the Rembrandt database were classified three groups; 46 FoxM1^high^, 92 FoxM1^mid^, 46 FoxM1^low^ GBMs. Overall survivals of these groups were compared and plotted. (C) Statistical analysis to determine potential association between FoxM1 levels and other clinical parameters including age, KPS and gender. P-value is calculated by using Fisher’s exact test.
(PDF)

S1 Table Clinical information of GBM patients. (PDF)
