# Supplementary material for: FoxM1 Promotes Stemness and Radio-Resistance of Glioblastoma by Regulating the Master Stem Cell Regulator Sox2
Source: PLoS One. 2015 Oct 7;10(10):e0137703. doi: 10.1371/journal.pone.0137703 (PMC4596841; doi:10.1371/journal.pone.0137703)
Supplement: S1 Fig — NS07-448 cells were cultured under two different culture conditions (sphere and serum culture condition). (A) The GBM cells were forced differentiation in serum culture condition (5% FBS) and stained with anti-neural differentiation markers antibodies (anti-GFAP, O4, Tuj1, and NeuN) to check differentiation status of GBM cells. Scale bar = 50 μm. (B) Signal intensities were analyzed and compared. (PDF) [file pone.0137703.s002.pdf]

SUPPORTING INFORMATION S1

S1 Fig. Differentiation and neural cell marker expression of GBM cells. NS07-448 cells were cultured under two different culture conditions (sphere and serum culture condition). (A) The GBM cells were forced differentiation in serum culture condition (5% FBS) and stained with anti-neural differentiation markers antibodies (anti-GFAP, O4, Tuj1, and NeuN) to check differentiation status of GBM cells. Scale bar=50 mm. (B) Signal intensities were analyzed and compared.

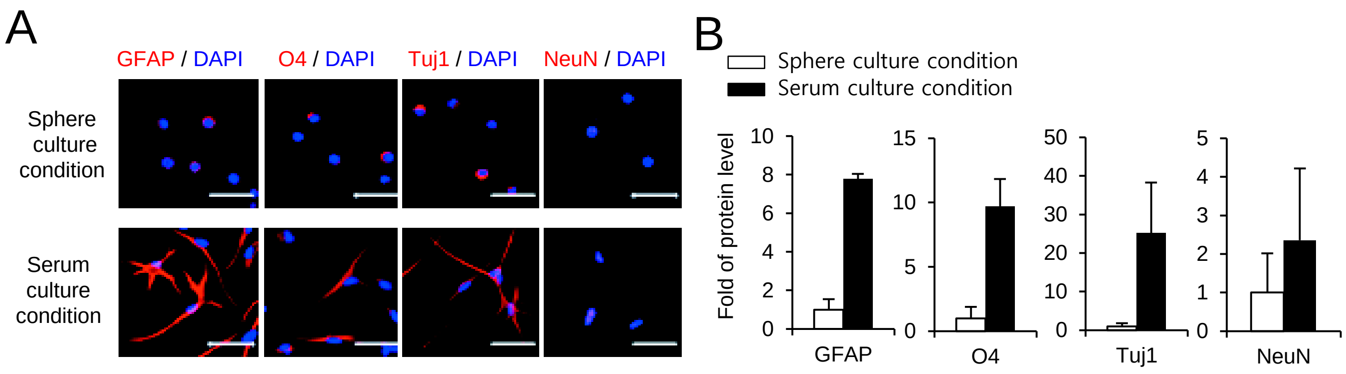

A
GFAP / DAPI
O4 / DAPI
Tuj1 / DAPI
NeuN / DAPI
Sphere culture condition
Serum culture condition
B
Sphere culture condition
Serum culture condition
Fold of protein level
GFAP
O4
Tuj1
NeuN
