# Supplementary material for: FoxM1 Promotes Stemness and Radio-Resistance of Glioblastoma by Regulating the Master Stem Cell Regulator Sox2
Source: PLoS One. 2015 Oct 7;10(10):e0137703. doi: 10.1371/journal.pone.0137703 (PMC4596841; doi:10.1371/journal.pone.0137703)
Supplement: S2 Fig — GBM11-096 GBM cells were infected with lentivirus carring FoxM1 shRNA. FoxM1 and Sox2 expressions were presented; *** and **, p<0.001 and <0.005 compared to NT shRNA, respectively. (PDF) [file pone.0137703.s003.pdf]

SUPPORTING INFORMATION S2

S2 Fig. Sox2 expression in FoxM1 knockdown cells. GBM11-096 GBM cells were infected with lentivirus carring FoxM1 shRNA. FoxM1 and Sox2 expressions were presented; *** and **, p<0.001 and <0.005 compared to NT shRNA, respectively.

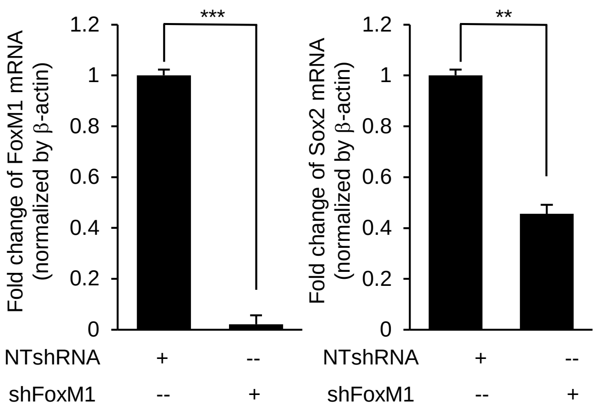
Fold change of FoxM1 mRNA
(normalized by β-actin)
1.2
1
0.8
0.6
0.4
0.2
0
***
NTshRNA
+
--
shFoxM1
--
+
Fold change of Sox2 mRNA
(normalized by β-actin)
1.2
1
0.8
0.6
0.4
0.2
0
**
NTshRNA
+
--
shFoxM1
--
+
