# Supplementary material for: FoxM1 Promotes Stemness and Radio-Resistance of Glioblastoma by Regulating the Master Stem Cell Regulator Sox2
Source: PLoS One. 2015 Oct 7;10(10):e0137703. doi: 10.1371/journal.pone.0137703 (PMC4596841; doi:10.1371/journal.pone.0137703)
Supplement: S3 Fig — FoxM1 mRNA expression in gliomas were derived from the public by Rembrandt database, which harbors 21 normal brain, 92 low grade gliomas, 69 anaplastic astrocytomas, and 126 GBM specimens, respectively. (B) GBM patients from the Rembrandt database were classified three groups; 46 FoxM1high, 92 FoxM1mid, 46 FoxM1low GBMs. Overall survivals of these groups were compared and plotted. (C) Statistical analysis to determine potential association between FoxM1 levels and other clinical parameters including age, KPS and gender. P-value is calculated by using Fisher’s exact test. (PDF) [file pone.0137703.s004.pdf]

SUPPORTING INFORMATION S3

S3 Fig. Clinical implications of FoxM1expression in Glioma patients. FoxM1 mRNA expression in gliomas were derived from the public by Rembrandt database, which harbors 21 normal brain, 92 low grade gliomas, 69 anaplastic astrocytomas, and 126 GBM specimens, respectively. (B) GBM patients from the Rembrandt database were classified three groups; 46 $FoxM1^{high}$, 92 $FoxM1^{mid}$, 46 $FoxM1^{low}$ GBMs. Overall survivals of these groups were compared and plotted. (C) Statistical analysis to determine potential association between FoxM1 levels and other clinical parameters including age, KPS and gender. P-value is calculated by using Fisher’s exact test.

A

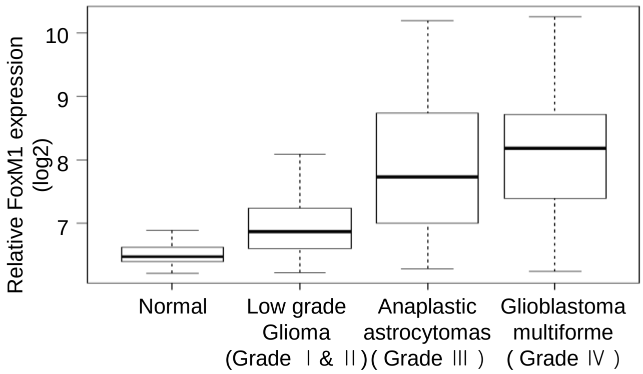

B

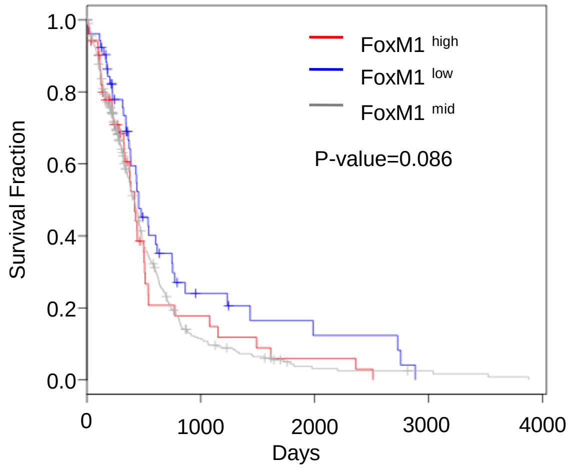

C

Group	Age		Average	Fisher's Test
	<=50	>50		
FoxM1 high	21	32	52.26415	1
FoxM1 low	20	32	53.38462	

Group	KPS		Average	Fisher's Test
	<=70	>70		
FoxM1 high	13	24	73.51351	0.6324487
FoxM1 low	12	29	76.34146	

Group	Gender		Average	Fisher's Test
	Male	Female		
FoxM1 high	33	20	NA	0.408948
FoxM1 low	37	15	NA	
