# Supplementary material for: FoxM1 Promotes Stemness and Radio-Resistance of Glioblastoma by Regulating the Master Stem Cell Regulator Sox2
Source: PLoS One. 2015 Oct 7;10(10):e0137703. doi: 10.1371/journal.pone.0137703 (PMC4596841; doi:10.1371/journal.pone.0137703)
Supplement: S1 Table — (PDF) [file pone.0137703.s005.pdf]

SUPPORTING INFORMATION S4

S4 Table. Clinical information of GBM patients

ID	IRS	Age	Sex M;1_F;2	Recurr Y;1_N;0	PFS	Death Y;1_N;0	OS
GBM 1	6	77	2	1		1	316
GBM 2	2	53	1	1	240	1	546
GBM 3	2	53	2	1	390	1	604
GBM 4	2	47	1	1	660	1	810
GBM 5	0	58	1	0	150	1	351
GBM 6	4	74	1	1	90	1	259
GBM 7	2	44	2	0		1	828
GBM 8	3	56	1	0	780	0	931
GBM 9	0	66	1	1	630	1	730
GBM 10	8	32	1	1	120	1	412
GBM 11	8	46	2	0	150	1	372
GBM 12	8	68	1	1	90	1	397
GBM 13	3	38	1	1	180	1	424
GBM 14	4	24	1	1	142	1	227
GBM 15	3	49	1	1	450	1	682
GBM 16	3	72	1	1		1	293
GBM 17	2	43	1		90		320
GBM 18	3	61	2		480		600
GBM 19	6	69	2	0	90	1	282
GBM 20	6	70	2	1	120	1	341
GBM 21	4	63	1	1	90	1	365
GBM 22	2	67	1	1	300	0	691
GBM 23	2	54	2				60
GBM 24	9	46	2	1	210	1	399
GBM 25	12	48	2	1	360	1	474
GBM 26	6	64	2				177
GBM 27	3	49	1	1	540	0	634
GBM 28	0	71	1	1		1	332
GBM 29	4	61	1				120
GBM 30	1	15	1	0	221	1	292
GBM 31	12	78	1				234
GBM 32	12	67	2				22
GBM 33	0	62	1	1	120	1	429
GBM 34	3	41	2	1	180	1	303
GBM 35	3	59	2	0	450	0	475
GBM 36	12	54	1	0	240	0	504
GBM 37	4	19	1	1	210	1	300
GBM 38	1	64	1		90		244
GBM 39	6	39	2	0	270	0	372
GBM 40	3	25	1	1	270	0	381
GBM 41	1	49	1	0	300	0	358
GBM 42	3	33	1	0	270	0	318
GBM 43	9	36	1	0	240	0	301
GBM 44	8	42	1	0	210	0	316
GBM 45	12	55	2	0	90	0	294
GBM 46	1	66	1	0	210	0	325
GBM 47	12	56	2	0	180	0	280
GBM 48	0	54	1	0	180	0	290
GBM 49	8	69	2	0	210	0	300
GBM 50	0	72	2	1	114	0	255
GBM 51	4	63	2	1	90	1	266
GBM 52	6	62	2	0	234	0	234
GBM 53	12	62	1	0	180	0	188
